# High-throughput identification of G protein-coupled receptor modulators through affinity mass spectrometry screening†
†Electronic supplementary information (ESI) available. See DOI: 10.1039/c7sc04698g


**DOI:** 10.1039/c7sc04698g

**Published:** 2018-02-20

**Authors:** Shanshan Qin, Mengmeng Meng, Dehua Yang, Wenwen Bai, Yan Lu, Yao Peng, Gaojie Song, Yiran Wu, Qingtong Zhou, Suwen Zhao, Xiping Huang, John D. McCorvy, Xiaoqing Cai, Antao Dai, Bryan L. Roth, Michael A. Hanson, Zhi-Jie Liu, Ming-Wei Wang, Raymond C. Stevens, Wenqing Shui

**Affiliations:** a iHuman Institute , ShanghaiTech University , 201210 , Shanghai , China . Email: shuiwq@shanghaitech.edu.cn; b College of Pharmacy , Nankai University , 300071 , Tianjin , China; c The National Center for Drug Screening , The CAS Key Laboratory of Receptor Research , Shanghai Institute of Materia Medica , Chinese Academy of Sciences , 201203 , Shanghai , China . Email: mwwang@mail.shcnc.ac.cn; d School of Life Science and Technology , ShanghaiTech University , 201202 , Shanghai , China; e Department of Pharmacology , Chapel Hill School of Medicine , University of North Carolina , NC 27599 Chapel Hill , USA; f GPCR Consortium , CA92078 San Marcos , USA; g School of Pharmacy , Fudan University , 201203 , Shanghai , China

## Abstract

G protein-coupled receptors (GPCRs) represent the largest class of cell surface proteins and thus constitute an important family of therapeutic targets. Therefore, significant effort has been put towards the identification of novel ligands that can modulate the activity of a GPCR target with high efficacy and selectivity. However, due to limitations inherent to the most common techniques for GPCR ligand discovery, there is a pressing need for more efficient and effective ligand screening methods especially for the identification of potential allosteric modulators. Here we present a high-throughput, label-free and unbiased screening approach for the identification of small molecule ligands towards GPCR targets based on affinity mass spectrometry. This new approach features the usage of target-expressing cell membranes rather than purified proteins for ligand screening and allows the detection of both orthosteric and allosteric ligands targeting specific GPCRs. Screening a small compound library with this approach led to the rapid discovery of an antagonist for the 5-HT receptor and four positive allosteric modulators for GLP-1 receptor that were not previously reported.

## Abstract 2

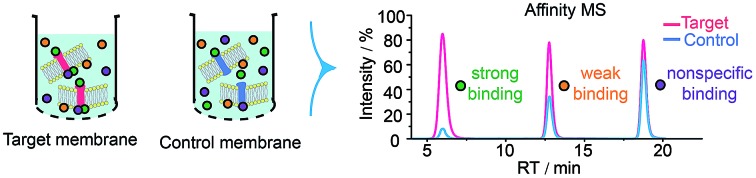
High-throughput identification of GPCR modulators through affinity MS screening.

## Introduction

The superfamily of G-protein-coupled receptors (GPCRs) is the largest class of cell surface receptors and they play a central role in a variety of pathophysiological conditions.^1^ GPCRs are recognized as an important family of therapeutic targets upon which an estimated 30–40% of marketed drugs act.^2^ While much effort has gone into identifying novel ligands that can modulate the activity of a GPCR target with high efficacy and selectivity, conventional techniques for GPCR drug discovery remain subject to several critical limitations. For example, receptor functional assays, which measure GPCR downstream signaling effectors,^3^ are inadequate for identifying allosteric or biased signaling modulators and often generate hits unsuitable for subsequent optimization.^4^ Radioligand binding assays, which assess receptor–ligand interactions on cell surfaces, are increasingly restricted due to high production costs and hazards to human health.^3^ Alternative receptor binding assays using fluorescently labeled probes require careful compound design and optimization because of the impact of fluorophore attachment on ligand affinity and efficacy.^5^,^6^ Finally, while surface plasma resonance and NMR have recently been employed in the identification of GPCR ligands,^7^–^9^ they require highly purified and stabilized receptors, which are not feasible for a number of targets and their current throughput is not amenable to large-scale compound library screening.

Affinity mass spectrometry (MS) has emerged as a powerful approach for analyzing protein–ligand interaction and it plays a vital role in early-phase drug discovery.^10^–^12^ In a typical affinity MS-based workflow, the ligand–bound protein complexes are first separated from unbound compounds by ultrafiltration or size exclusion chromatography. Then the ligands dissociated from the complexes are identified by LC-MS/MS analysis.^10^,^11^,^13^,^14^ Similar to other biophysical approaches, affinity MS has been widely applied to ligand identification for purified protein targets from compound libraries.

Affinity MS-based assays have been developed for screening chemical ligands towards different soluble protein targets, especially enzymes and kinases of therapeutic values.^10^,^12^,^13^,^15^–^17^ However, the application of affinity MS techniques to ligand discovery for membrane receptors is substantially hampered due to the difficulty of obtaining membrane proteins of sufficient purity, activity and stability. Whitehurst *et al.* first showcased the adaptation of affinity MS to screening ligands towards the membrane receptor CXCR4 that belongs to the GPCR family.^18^ To search for an optimal form of the receptor for screening purposes, the authors laboriously compared different epitope tags and detergents to find the best conditions for expression and purification of the receptor. They argued that sufficient yield and purity of the receptor is essential for successful usage of this screening approach.^18^ However, it is widely recognized that many transmembrane receptors are unstable when isolated away from the cell membrane. Thus, biophysical techniques that can only analyze purified proteins such as isothermal titration calorimetry (ITC), surface plasmon resonance (SPR) as well as affinity MS approach are not amenable to many receptors that are attractive drug targets.

Here we developed a novel affinity MS technique that enables ligand screening towards wild-type active receptors embedded in the cell membranes. Most significantly, the challenging and laborious receptor purification step is eliminated in our workflow. We implemented this new approach to achieve high-throughput, label-free and unbiased ligand screening towards two GPCR targets, which resulted in the discovery of unreported orthosteric ligands and allosteric modulators for specific GPCRs.

## Results and discussion

**Fig. 1:**
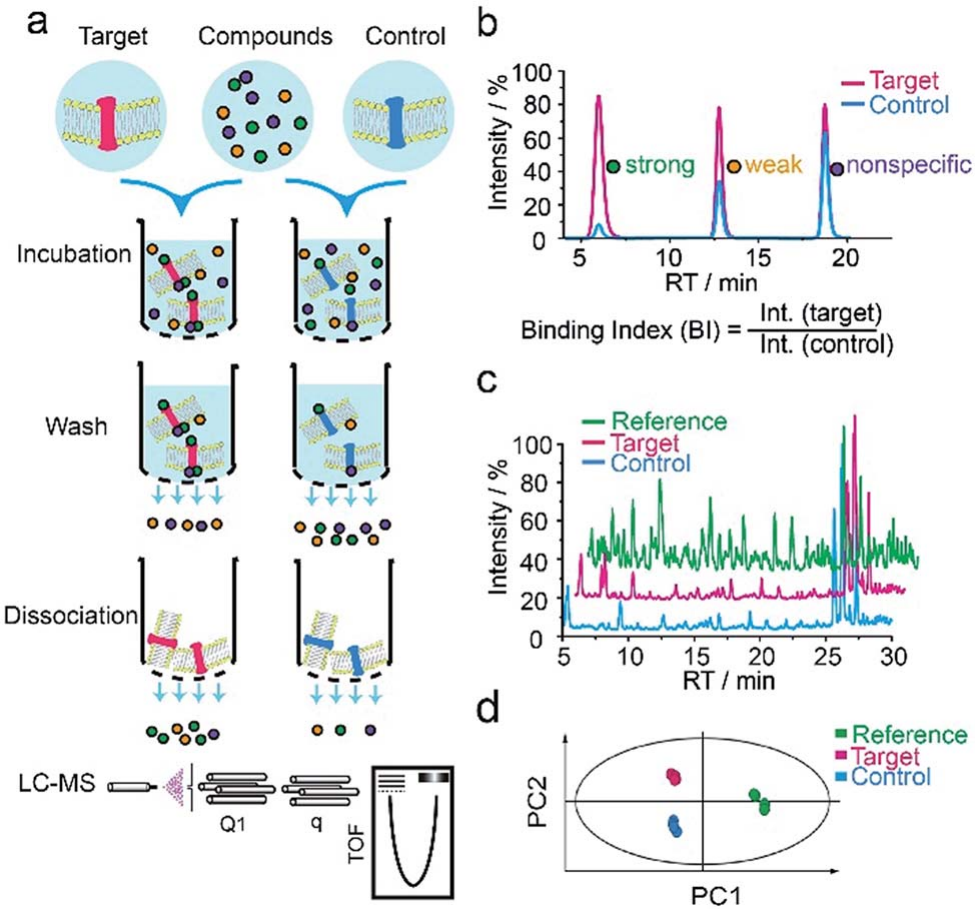
GPCR ligand identification through affinity MS screening. (a) Experimental workflow. (b) BI derived from LC-MS measurement reflects the relative binding affinity of each compound to the target. (c) Representative LC-MS chromatograms of cocktail mix-1 (reference), compounds from the target incubation (target) and from control incubation (control). (d) Multivariate analysis of the LC-MS data with an OPLS-DA model.^17^

We first applied our methodology using the human 5-hydroxytryptamine 2C receptor (5-HT_2C_R), an anti-psychotic drug target for treating depression, schizophrenia and other mental disorders.^19^,^20^ The membrane fractions from insect cells expressing 5-HT_2C_R were directly incubated with a cocktail of compounds while the protein concentration was kept in large excess over any compound (see ESI†). Cell membranes were separated from the compound solution by filtration. Compounds associated with the receptor-expressing membranes were released after washing and subjected to liquid chromatography coupled to high-resolution mass spectrometry (LC-MS) analysis (Fig. 1a). Insect cell membranes expressing rhodopsin were prepared in the same manner and used as a negative control (Fig. S1†). To assess the specific association of a given compound with the receptor, we calculated a binding index (BI) defined as the ratio of MS response of the compound detected in the target *versus* the control incubations (Fig. 1b). This BI parameter allowed us to compare the affinities of different compounds bound to the GPCR target and discern compounds non-specifically interacting with the cell membranes.

To validate our method, we incubated the 5-HT_2C_R-expressing membranes with a mixture of 5 known ligands to the receptor and 45 unrelated compounds. Given all positive ligands with *K*_i_ 0.3–29 nM showing BI > 2 (Fig. S2†) as well as our previous experience in selecting ligands to soluble protein targets with affinity mass spectrometry,^15^–^17^ we defined a BI threshold of 2 for distinguishing putative ligands to the receptor from non-binders. Next, we employed the affinity MS assay to screen a collection of 4333 small molecules against 5-HT_2C_R. This library was divided into 9 cocktails (each containing 480 or 493 compounds) and each was separately incubated with the target-expressing and control membranes in quadruplicate. Representative LC-MS chromatograms are shown in Fig. 1c. Multivariate analysis^17^ of the LC-MS data from reference, target and control revealed that the composition of compounds associated with 5-HT_2C_R was substantially deviated from that in the mix-1 reference and the control (Fig. 1d).

**Fig. 2:**
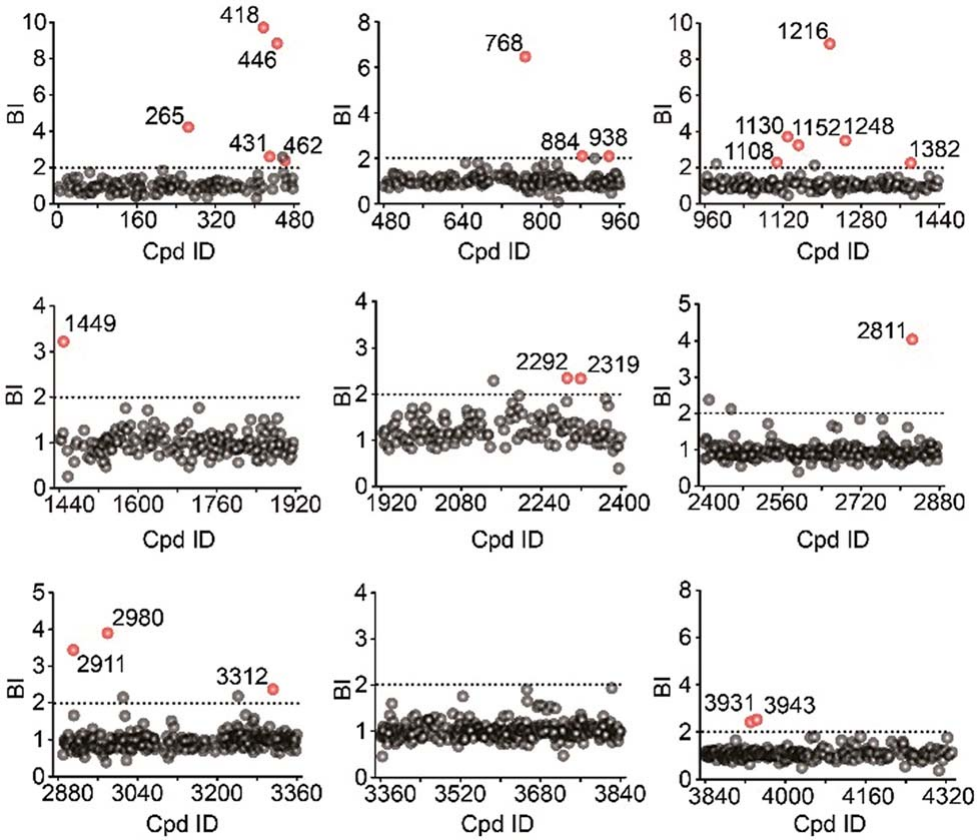
Affinity MS screening of a 4333-member compound library split into 9 cocktails against 5-HT_2C_R. Initial hits (mean BI > 2 and *p* < 0.05) are indicated by red dots while grey dots represent negatives.

Using stringent data processing criteria for compound annotation and hit selection (see ESI†), we identified a total of 23 initial hits from screening the 9 cocktails (Fig. 2, full data set in Table S1†). Twelve were well characterized antagonists for 5-HT_2C_R or a closely-related subfamily member 5-HT_2A_R with a mean BI of 2.16 to 9.71 (full data set in Table S2†). Most of these known ligands showed <10 nM binding affinity to the receptor as determined by the radioligand competition assay. Results from a thermal shift assay using purified 5-HT_2C_R indicated that 11 known ligands could significantly enhance the receptor's thermostability thereby validating their direct interactions with the receptor. Notably, 98 compounds in our collection that were reported to be 5-HT_2C_R ligands had BI < 2 from the primary screening. While their IC_50_ or *K*_i_ values from the ChEMBL database were all above 10 nM, 85% had IC_50_ or *K*_i_ > 100 nM towards 5-HT_2C_R. Therefore, the stringent BI cut-off defined for hit selection ensured identification of highest affinity ligands with a minimal false-positive rate.

**Fig. 3:**
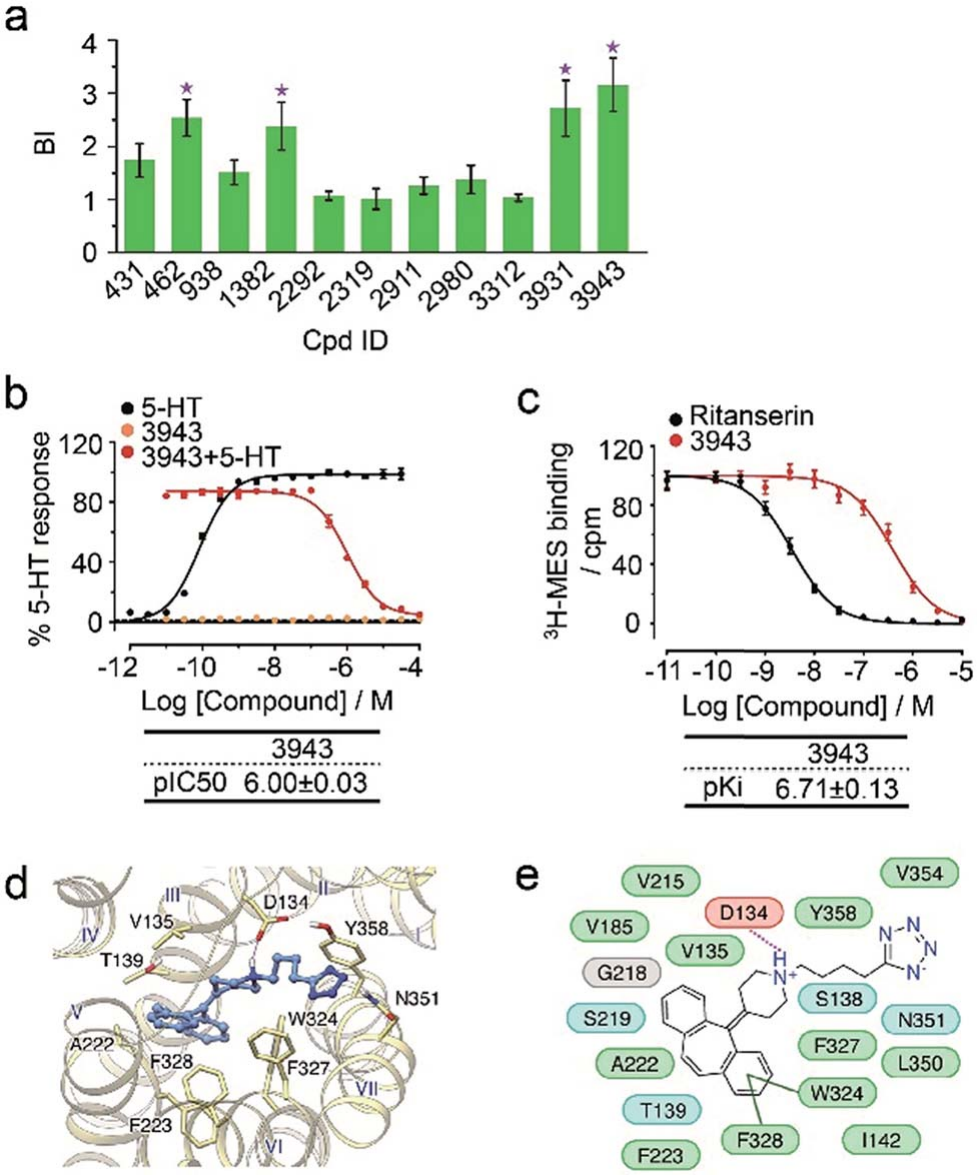
(a) Hit validation with the affinity MS assay on a mixture of the 11 putative ligands. Confirmed ligands (mean BI > 2 and *p* < 0.01) are designated by asterisks, with data shown as mean and s.d. of four individual assays. (b) Affinity of 3943 to 5HT_2C_R determined by radioligand binding assay. (c) Inhibition of 5HT_2C_R activation by 3943 revealed by calcium mobilization assay. MES, mesulergine. *K*_i_ and IC_50_ measurement in (b) and (c) were represented by mean and s.d. from experimental triplicates. (d) Docked poses of 3943 (blue) in the 5-HT_2C_R model (yellow). (e) Schematic of the interactions between 3943 and 5-HT_2C_R. Residue colors: negatively charged, red; hydrophobic/aromatic, green; polar, cyan; glycine, gray. Interactions: hydrogen bonds to the side chain, dashed magenta line; hydrogen bonds to the main chain, solid magenta line; π–π interaction, dark green line.

To verify the 11 initial hits with no documented binding to the 5-HT_2_ receptor family, a simple mixture was created by pooling and incubating them with the receptor-expressing membranes. Four hits were verified in this secondary affinity MS assay (Fig. 3a). Those that were invalidated could have resulted from compound misidentification or altered binding properties in the original complex mixtures. The unknown ligand 3943 had a thermostabilizing effect on the purified receptor (Table S3†) and it further exhibited moderate antagonist activity in the calcium mobilization assay as it inhibited the 5-HT induced activation of 5-HT_2C_R with IC_50_ of 1.01 μM (Fig. 3b). Notably, this compound displayed even stronger antagonism against 5-HT_2A_R (IC_50_ = 0.12 μM) and 5-HT_2B_R (IC_50_ = 0.51 μM) (Fig. S3a†). The radioligand competition assay verified potent antagonist binding of 3943 to all three 5-HT_2_ receptor subtypes in the nanomolar range, with stronger affinity to 5-HT_2A/2B_R than 5-HT_2C_R (Fig. 3c, S3b†). It was not surprising to identify a new antagonist against three 5-HT_2_ receptor subfamily members using our approach because of the high sequence homology and very similar ligand binding pockets among them. A molecular docking study demonstrated key interactions between the cyproheptadine scaffold in 3943 and conserved residues in the transmembrane helices III, V, VI and VII of 5-HT_2A/2B/2C_ receptors (Fig. 3d and e).

Next we applied our affinity MS assay to a more challenging target, the human glucagon-like peptide-1 receptor (GLP-1R). GLP-1R is a class B GPCR that mediates the action of peptide hormone GLP-1 and exerts important functions in glucose homeostasis.^21^,^22^ Although small molecule modulators of GLP-1R are expected to serve as critical chemical tools for investigating ligand-directed biased signaling, very few non-peptidic GLP-1R agonists have been published.^21^ Before embarking on a real library screening, we first optimized our approach using two negative allosteric modulators (NAMs) of the receptor, PF06372222 and NNC0640. Both NAMs were readily identified from a 50-compound mixture with the mean BI of 16.66 for PF06372222 and 3.31 for NNC0640, indicating their significant association with the receptor in the membrane fraction (Fig. S4†).

**Fig. 4:**
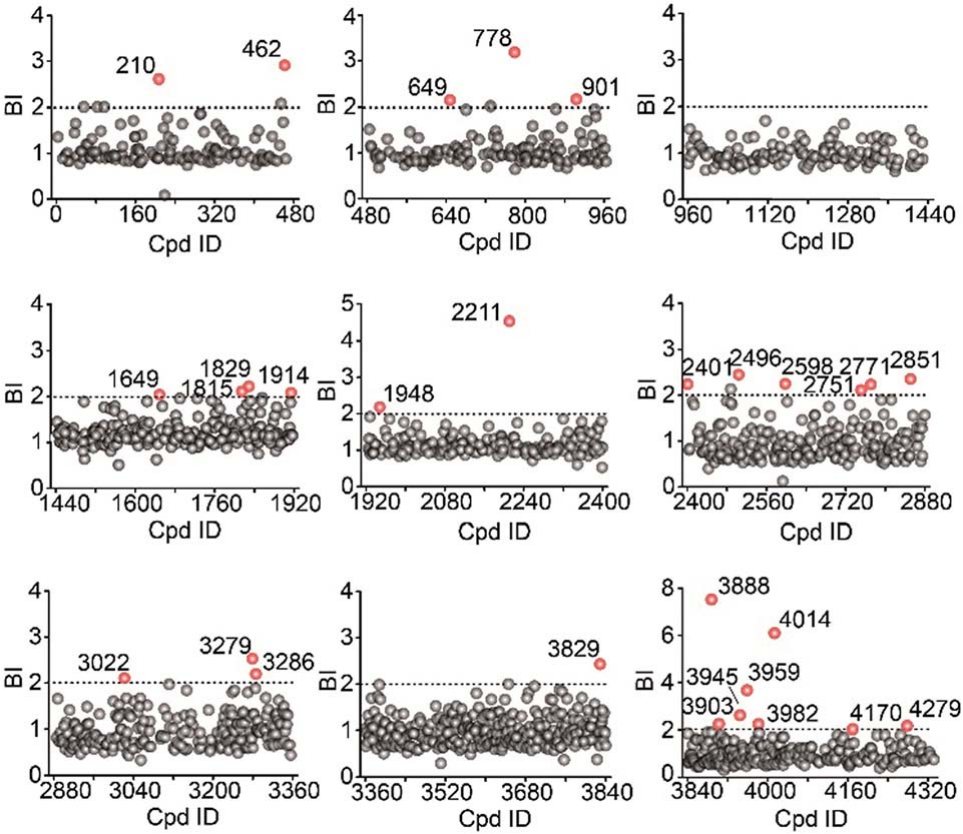
Affinity MS screens of the 9 cocktails from the above compound library against GLP-1R. Initial hits (mean BI > 2 and *p* < 0.05) are indicated by red dots with grey dots representing negatives.

We then employed the affinity MS approach to screen the previous nine compound cocktails using insect cell membranes expressing the GLP-1R transmembrane domain (TMD). A total of 29 putative ligands were obtained from the primary screening (Fig. 4, full date set in Table S4†), and none of them have been previously linked with GLP-1R.

**Fig. 5:**
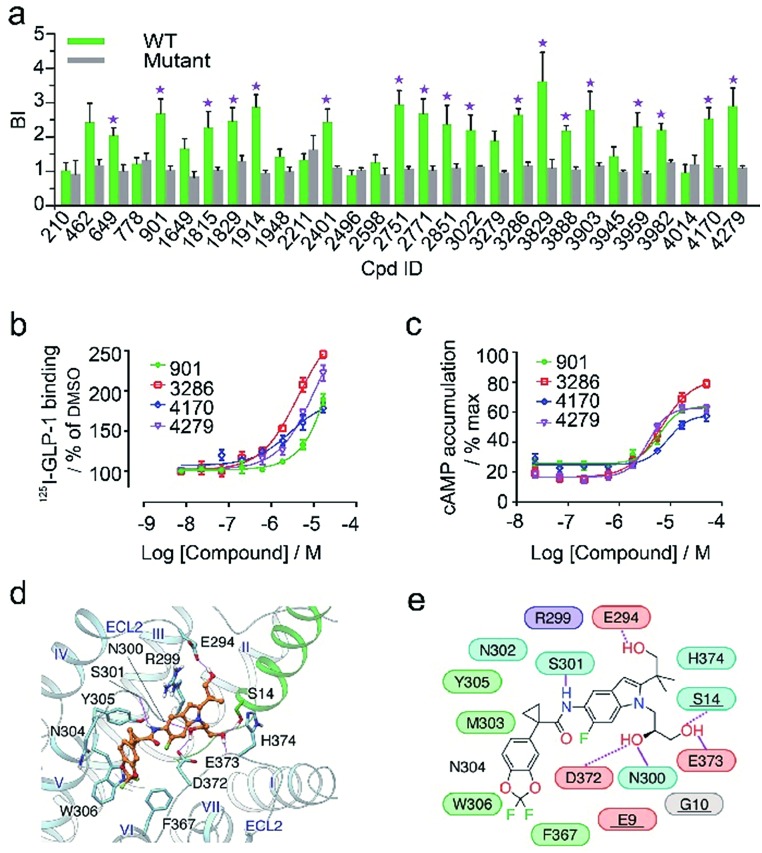
(a) Affinity MS assay on a mixture of 29 initial hits using wild-type (WT) receptor-expressing membranes or point-mutated receptor-expressing membranes. Confirmed ligands (mean BI > 2 and *p* < 0.01) are designated by asterisks with data shown as mean and s.d. of four individual assays. (b) Affinity of each test ligand to GLP-1R determined by radioactive GLP-1 binding assay. (c) Enhanced GLP-1R cell activity in the presence of GLP-1 (0.02 nM) by each test ligand. pEC50 measurement for (b) and (c) is provided in Table S5.† (d) Docked pose for 3286 (orange) in the GLP-1 (green)/GLP-1R (light blue) complex model. (e) Schematic representation of interactions between 3286 and GLP-1R. Residues of GLP-1 are underlined. Color codes for residues and interactions are as in Fig. 3e. While the predicted binding site is formed by extracellular loop 2 (ECL2), extracellular loop 3 (ECL3), transmembrane helix VI and GPL-1, 3268 seems to strengthen the interactions between ECL2, ECL3 and GLP-1, which may explain its PAM activity in the presence of GLP-1.

In the secondary affinity MS screening assay, 18 were confirmed to bind the receptor (Fig. 5a). Importantly, in this step we also prepared cell membranes expressing another GLP-1R TMD construct with stabilizing mutations commonly exploited in the GPCR crystallography.^23^,^24^ Interestingly, all ligands identified in our primary screening abrogated their binding to the thermostabilized receptor (Fig. 5a). This result highlighted the unique advantage of using cell membranes expressing wild-type receptors in ligand screening given that purified receptors with mutations could be locked in an inactive conformation and fail to engage ligands that only interact with the active receptor. Subsequent radioligand binding assay revealed that four ligands (901, 3286, 4170, and 4279) augmented peptide binding to the receptor with EC_50_ in the low micromolar range (Fig. 5b, Table S5†). When conducting another binding assay using radiolabeled exendin-4_(9-39)_, which is a GLP-1R antagonist targeting the extracellular domain (ECD), we observed no obvious alteration of the binding potency between exendin-4_(9-39)_ and GLP-1R by any of the four ligands (Fig. S5a†). Moreover, increasing the amount of NAM PF06372222 in the presence of the four ligands did not compete off any ligand in the affinity MS assay (Fig. S5b†).

These results collectively imply that the four novel ligands allosterically modulate the binding of GLP-1 to the orthosteric pocket of GLP-1R and that their targeting sites are likely to be different from the NAM binding pocket.^25^ Concordantly, all four ligands substantially promoted intracellular cAMP accumulation in the presence of GLP-1 with EC_50_ in the low micromolar range (Fig. 5c, Table S5†). By contrast, none of them stimulated cAMP production in cells expressing glucagon receptor (GCGR), a close homolog of GLP-1R, demonstrating the high selectivity of the four ligands for GLP-1R (Fig. S5c†). Therefore, we conclude that the four ligands identified in this study are all positive allosteric modulators (PAMs) of GLP-1R. For compound 3286, we performed molecular docking based on a previous model of the GLP-1/GLP-1R complex^26^ to reveal a possible binding mode of this PAM (Fig. 5d and e). While the precise targeting sites and activation mechanism of the four PAMs warrant further investigation, we are excited by the discovery of a new class of ligands for this important therapeutic target and the efficiency of our approach in identification of novel GPCR ligands.

A notable limitation of our approach lies in the detectability issue for certain compounds by our LC-MS analytical platform. In our study, the percentage of detectable compounds in each library cocktail by electrospray MS in the positive mode varied between 66–86% (Fig. S6†). Thus, the receptor binding capability of the undetectable compounds remains unclear. This could be partially addressed using MS instruments of increased sensitivity and operating in both positive and negative modes, or by optimizing the LC system for effective separation of abnormal compounds. Nevertheless, our current LC-MS method is generally applicable to the analysis of most drug-like small molecules.

## Conclusions

In summary, we developed an efficient and convenient affinity MS approach for high-throughput identification of GPCR ligands. Our new approach demonstrates five major advantages over the conventional receptor functional assays or ligand binding assays: (1) both the receptor and compounds are label-free; (2) the receptor target, with minimal sequence modification, is embedded in the cell membranes to retain its native conformation during ligand interaction; (3) measurement of direct receptor–ligand binding in an unbiased manner facilitates identification of allosteric modulators targeting uncharacterized binding sites; (4) quantitative comparison of ligand response with a GPCR control enables identification of specific ligands with medium affinity while maintaining a low false-positive rate; and (5) protein purification commonly required in affinity-based ligand screens is unnecessary, thus reducing experimental cost and eliminating purification-inherent drawbacks. Our screening technology, benchmarked on two GPCR targets, can be readily adapted to other membrane receptors. Its integration with complementary biophysical and functional assays could expedite the discovery of new GPCR modulators with therapeutic potential. Apart from direct application to drug discovery towards membrane receptors, our approach is expected to aid in delineating the membrane protein–small molecule interaction network within the cell.

## Experimental

### Preparation of cell membranes over-expressing 5HT_2C_-R or GLP1-R TMD

The 5-HT_2C_R construct was produced comprising residues 40–393 with residues from intracellular loop 3 (245–301) replaced by thermo-stabilized *E. coli* apocytochrome b_562_RIL (BRIL). It was expressed with an N-terminal FLAG tag and a C-terminal 10× His tag. The GLP-1R TMD construct comprised residues 128–431 with residues from intracellular loop 2 (258–263) replaced by rubredoxin and it was expressed with an N-terminal FLAG tag followed by BRIL and a C-terminal 10× His tag. The rhodopsin construct comprised residues 2–331 with residues from intracellular loop 3 (235–241) replaced by BRIL and it was expressed with an N-terminal FLAG tag and a C-terminal 10× His tag. All three proteins were expressed using the Bac-to-Bac Baculovirus Expression System (Invitrogen) in *Spodoptera frugiperda* (*Sf*9) cells. Cells were infected at a density of 2–3 × 10^6^ cells per mL with baculovirus at a multiplicity of infection (MOI) of 5. Cultures were grown at 27 °C and harvested 48 h after infection. Cell pellets were lysed by repeated washing and ultra-centrifugation in the hypotonic buffer of 10 mM HEPES (pH 7.5), 10 mM MgCl_2_, 20 mM KCl, and the high osmotic buffer of 10 mM HEPES (pH 7.5), 1.0 M NaCl, 10 mM MgCl_2_, 20 mM KCl, both with EDTA-free protease inhibitor cocktail tablets (Roche). The washed membranes were re-suspended in the hypotonic buffer with 30% glycerol and stored at –80 °C for further usage. Cell membrane proteins were extracted using 1% SDS in 0.1% NaOH and total protein concentration was measured with the BCA quantification kit (TIANGEN, China). The amount of 5-HT_2C_R and GLP-1R present in the membrane extract was determined using ELISA with anti-His antibody (Genscript, China).

### Compound library preparation

The library of 4333 small molecule members was purchased from Topscience (Shanghai, China) in DMSO stock. It was divided into nine cocktails (mix-1 to mix-9) by pooling different sub-fractions of the library. Eight cocktails (mix-1 to mix-8) comprised 480 compounds and the last one (mix-9) comprised 493 compounds. No compounds were overlapped between different cocktails. All cocktails were stored at –20 °C.

### GPCR ligand identification with affinity mass spectrometry

#### Sample preparation for screening and hit validation

In the primary screening, the membrane fraction expressing the receptor (5HT_2C_R or GLP-1R TMD) was incubated with a compound cocktail in a buffer of 10 mM HEPES (pH 7.5), 10 mM MgCl_2_ and 20 mM KCl in a total volume of 200 μL for 1 h at 25 °C. During incubation, each compound concentration was at 50 nM and the receptor concentration was estimated to be 200–250 nM. The membrane fraction was separated from the incubation solution by rapid vacuum filtration through MultiScreenHTS FB Filter Plate (Millipore) using a MultiScreenHTS vacuum Manifold (Millipore). After washing the membrane fraction six times with ice-cold 150 mM ammonium acetate (pH 7.5), methanol was added to the membranes (100 μL, 4 times) and the filtrate, containing compounds initially associated with the membranes, was collected. We then used the Ostro™ 96-well plate (Waters) to deplete phospholipids co-eluted with the compounds of interest. The eluted samples were evaporated by a speed vacuum and reconstituted in 50% methanol before LC-MS analysis. The control sample was prepared by using rhodopsin-expressing membranes in incubation with the same amount of total membrane proteins as the target-expressing membranes. Each pair of target and control samples was prepared in quadruplicate.

In secondary screening, initial hits were pooled to make a simple mixture, which was incubated with target and control membranes separately under the same conditions. In the 5HT_2C_R experiment, 11 unknown ligands were pooled whereas in the GLP-1R experiment, all 29 initial hits were pooled. Compound concentration in incubation was still 50 nM and the receptor concentration was increased to 300 nM. In the method validation study, both target-expressing and control membranes were incubated with a 50-compound mixture containing specific known ligands and unrelated compounds with the same receptor and compound concentrations as mentioned above. Each pair of target and control samples was prepared in quadruplicate.

#### LC-MS analysis

Samples were analyzed on a Shimazu L30A UPLC system (Shimazu) coupled to a TripleTOF 6600 mass spectrometer (AB SCIEX) operating in the positive ion mode. Chromatographic separation was performed on a ZORBAX Eclipse Plus C18 column (3.5 μm, 2.1 × 100 mm, Agilent) at a flow rate of 400 μL min^–1^ and maintained at 40 °C with the mobile phases of water/0.1% formic acid (A) and acetonitrile/0.1% formic acid (B). In the primary screening, the LC gradient was as follows: 0–2 min, 5% B; 2–2.1 min, 5–20% B; 2.1–22 min, 20–35% B; 22–30 min, 35–60% B; 30–30.5 min, 60–90% B; 30.5–35 min, 90% B; and re-equilibration for 5 min. In the secondary screening, a shorter LC gradient was applied: 0–2 min, 5% B; 2–2.1 min, 5–20% B; 2.1–10 min, 20–35% B; 10–13 min, 35–60% B; 13–13.5 min, 60–90% B; 13.5–16 min, 90% B; and re-equilibration for 4 min. Full-scan mass spectra were acquired in the range of 100–1500 *m*/*z* with major ESI source settings: voltage 5.0–5.5 kV; gas temperature 500 °C; curtain gas 35 psi; nebulizer gas 55 psi; and heater gas 55 psi. MSMS spectra were acquired on selected compound precursors with collision energy set at 45 eV with a CE spread of 15 eV and other ion source conditions identical to MS full scans. For each sample set, we first injected a reference sample (the compound mixture alone) followed by four pairs of target and control samples.

#### LC-MS data processing and hit selection

First, compounds in the reference were identified by extracting selected ion chromatograms (EICs) using Peakview 2.2 (AB SCIEX) based on accurate mass measurement (<10 ppm deviation) and isotope envelop matching (<20% difference from the theoretical envelop). H^+^ and Na^+^ adducts were considered for compound detection. Then compounds in target and control samples were identified by meeting the above criteria plus retention time (RT) matching with corresponding peaks in the reference (<0.2 min shift). Ambiguous peaks of isomeric compounds in the cocktail were distinguished by acquiring MS/MS spectra or injecting individual standards for RT differentiation. For each compound confidently identified in target and control samples, its BI was calculated by dividing the EIC intensity of the compound detected in the target sample by that in the control. Given that target and negative control proteins were expressed at similar levels and their concentrations during ligand incubation were close to each other (<20% difference), we did not modify the BI ratios with the protein concentration ratios. Initial hits were selected based on a mean BI > 2 and *p* < 0.05 from four replicates. Hits were validated in the second-round MS affinity assay based on a mean BI > 2 and *p* < 0.01 from four replicates. *P*-values were determined by a two-tailed *t*-test of BI values against unity. Putative ligands were searched in ChEMBL, DrugBank, Binding DB and SciFinder databases to find out whether they are known ligands to the receptor target.

### Purification of 5-HT_2C_R protein for the thermal shift assay

The receptor protein was extracted from the previously purified membranes by adding *n*-dodecyl-d-maltopyranoside (DDM, Affymetrix) and cholesteryl hemisuccinate (CHS, Sigma) to the membrane suspension to a final concentration of 1.0% (w/v) and 0.2% (w/v), respectively, in buffer of 50 mM HEPES (pH 7.5) and 150 mM NaCl, while stirring continuously at 4 °C for 2 h. The supernatant was isolated by centrifugation at 160 000*g* for 30 min followed by overnight incubation in TALON IMAC resin (Clontech) at 4 °C. The resin was washed with ten column volumes of wash buffer 1 (50 mM HEPES, pH 7.5, 800 mM NaCl, 0.1% (w/v) DDM, 0.02% (w/v) CHS, 20 mM imidazole, 10% (v/v) glycerol) and followed by five column volumes of wash buffer 2 (50 mM HEPES, pH 7.5, 150 mM NaCl, 0.05% (w/v) DDM, 0.01% (w/v) CHS, 10% (v/v) glycerol). Proteins were eluted in 5 column volumes of wash buffer 2 with 250 mM imidazole. Protein purity and monodispersity were assessed by SDS-PAGE and analytical size-exclusion chromatography.

The thermal shift assay using a CPM fluorescent dye (Sigma) was performed as described in the literature.^27^ Briefly, purified receptor protein pre-mixed with the CPM dye was incubated with a given compound at 200 μM at 4 °C for 1 h. The protein sample was heated step-wise on a Rotor-Gene Thermo-optical Analyzer (QIAGEN Gmbh) from 25 °C to 95 °C. Upon temperature rise, the proteins unfolded and cysteine residues were exposed to form adducts with CPM, which were detected by fluorescence at 387/463 nm using an EnVision multilabel plate reader (PerkinElmer). From the normalized thermal stability curve, melting temperatures (*T*_m_) were obtained by fitting the curve with a Boltzmann sigmoidal function using GraphPad Prism. Comparison of *T*_m_ for the apo receptor and receptor incubated with a compound gave rise to *T*_m_ shift (Δ*T*_m_) for the compound.

### Calcium mobilization assay

HEK293T cells stably transfected with 5-HT_2A/2B/2C_ receptor were seeded in 384-well plates at a density of 15 000 cells per well in DMEM containing 1% dialyzed FBS 8 h before assaying. After removing the medium, cells were incubated (20 μL per well) for 1 h at 37 °C with Fluo-4 Direct dye (Invitrogen) and reconstituted in a FLIPR buffer (1× HBSS, 2.5 mM probenecid, and 20 mM HEPES, pH 7.4). After the dye loaded, cells were placed in a FLIPR TETRA fluorescence imaging plate reader (Molecular Devices). Drug dilutions, prepared at 3× final concentration in FLIPR buffer and aliquoted into 384-well plates, were also added to the FLIPR TETRA. The fluidics module and plate reader of the FLIPR TETRA were programmed to read baseline fluorescence for 10 s (1 read per s), then to add 10 μL of drug per well and to read for 6 min (1 read per s). Fluorescence in each well was normalized to the average of the first 10 reads (*i.e.*, baseline fluorescence). Then the maximum-fold increase, which occurred within 60 s after drug addition over baseline fluorescence elicited by vehicle or a test compound, was determined. In the test of potential positive allosteric modulators and antagonists, 5-HT at EC_20_ (0.1 nM) and at EC_80_ (3 nM) were added to the medium, respectively, for receptor activation.

### cAMP accumulation assay

The human GLP-1R and glucagon receptor (GCGR) cDNAs were cloned into pcDNA3.1/V5-His-TOPO (Invitrogen). After 24 h transfection, stably expressing CHO-K1 cells were selected on 750 mg mL^–1^ G418 (Roche) for 2 weeks to obtain the clone with the highest expression and potent cell activity. Stable cells were seeded in 6-well plates for overnight culture before inoculation into 384-well plates (8000 cells per well) for the assay. Accumulation of cAMP was measured using the LANCE cAMP kit (PerkinElmer) according to the manufacturer's instructions. Briefly, cells were incubated for 30 min in assay buffer (DMEM, 1 mM 3-isobutyl-1-methylxanthine) with varying concentrations of each compound (2.3 nM to 50 μM) in the presence of GLP-1 or glucagon (0.02 nM) at 37 °C. Compound treatment was quenched by adding lysis buffer containing LANCE reagents. Plates were then incubated for 60 min at room temperature and time-resolved FRET signals were measured at 620 nm and 650 nm by an EnVision multilabel plate reader (PerkinElmer).

### Radioligand binding assays for different receptors

The 5-HT_2A/2B/2C_R binding assays were provided by the National Institute of Mental Health Psychoactive Drug Screening Program (NIMH PDSP), contract number HHSN-271-2008-00025-C. Detailed procedures are available at ; https://pdspdb.unc.edu/pdspWeb/. Briefly, 5-HT_2A/2B/2C_R membrane fractions were prepared from stable HEK293 cell lines or transient transfections. The radioligand used was ^3^H-ketanserin at 1–2 nM for 5-HT_2A_R; ^3^H-LSD at 1–2 nM for 5-HT_2B_R; ^3^H-mesulergine at 1–3 nM for 5-HT_2C_R. Nonspecific binding was determined in the presence of 10 mM clozapine for 5-HT_2A_R, 10 mM SB206553 for 5-HT_2B_R, and 10 mM ritanserin for 5-HT_2C_R. Results were analyzed in GraphPad Prism with a built-in competitive binding equation to obtain affinity values.

In the GLP-1R and GCGR binding assays, stable cell lines were seeded onto 96-well poly-d-lysine treated cell culture plates (PerkinElmer) at a density of 3 × 10^4^ cells per well. Cells were harvested after 24 h post seeding, washed twice and incubated with blocking buffer (F12 supplemented with 33 mM HEPES, pH 7.4, and 0.1% bovine serum albumin (BSA)) for 2 h at 37 °C. They were then washed twice with PBS and incubated in binding buffer (PBS supplemented with 10% BSA, pH 7.4) with a test ligand at room temperature for 3 h. Cells were treated with each ligand at varying concentrations (7.6 nM to 16.7 μM) in the presence of ^125^I-GLP-1 (40 pM) or ^125^I-exendin-4_(9-39)_ (40 pM). Cells were then washed three times with ice-cold PBS and lysed by 50 μL lysis buffer (PBS supplemented with 20 mM Tris–HCl, 1% Triton X-100, pH 7.4). The plates were subsequently counted for radioactivity (counts per minute, CPM) in a scintillation counter (MicroBeta2TM Plate Counter, PerkinElmer) using a scintillation cocktail (OptiPhase SuperMix, PerkinElmer).

### Molecular modeling and docking

Binding mode prediction was performed using graphical user interface Maestro 10.4 in Schrödinger Suite 2015-4. For 5-HT_2C_R, a homology model of the receptor was built based on the crystal structure of 5-HT_2B_R^28^ (PDB: ; 4IB4) using the Advanced Homology Modeling tool. For GLP-1R, a previously published model^26^ was used and the allosteric binding site was identified with the SiteMap tool. Ligand 3D structures were generated using LigPrep 3.6. Molecular docking was carried out using Induced Fit Docking with the extra precision docking score and allowing optimization of residues within 5.0 Å.

## Conflicts of interest

There are no conflicts to declare.

## Supplementary Material

Supplementary information

Supplementary information

Supplementary information
