# Supplementary material for: High-throughput identification of G protein-coupled receptor modulators through affinity mass spectrometry screening
Source: Chem Sci. 2018 Feb 20;9(12):3192–9. doi: 10.1039/c7sc04698g (PMC5916221; doi:10.1039/c7sc04698g)
Supplement: Supplementary file 1 [file SC-009-C7SC04698G-s001.pdf]

Electronic Supplementary Material (ESI) for Chemical Science.
This journal is © The Royal Society of Chemistry 2018

Electronic Supplementary Material (ESI) for Chemical Science.
This journal is © The Royal Society of Chemistry 2017

Supporting Information

High-Throughput Identification of G Protein-Coupled Receptors Modulators through Affinity Mass Spectrometry Screening

Shanshan Qin,‡[a] Mengmeng Meng,‡[b] Dehua Yang,‡[c] Wenwen Bai,‡[b] Yan Lu,[ad] Yao Peng,[a] Gaojie Song,[a] Yiran Wu,[a] Qingtong Zhou,[a] Suwen Zhao,[ad] Xiping Huang,[e] John D. McCorvy,[e] Xiaoqing Cai,[c] Antao Dai,[c] Bryan L. Roth,[e] Michael A. Hanson,[f] Zhi-Jie Liu,[a,d] Ming-Wei Wang,*[c,d,g] Raymond C. Stevens[a,d] & Wenqing Shui*[a,d]

a, iHuman Institute, ShanghaiTech University, 201210, Shanghai, China

b, College of Pharmacy, Nankai University, 300071, Tianjin, China

c, The National Center for Drug Screening and the CAS Key Laboratory of Receptor Research, Shanghai Institute of Materia Medica, Chinese Academy of Sciences, 201203, Shanghai, China

d, School of Life Science and Technology, ShanghaiTech University, 201202, Shanghai, China

e, Department of Pharmacology, Chapel Hill School of Medicine, University of North Carolina, NC 27599 Chapel Hill, USA

f, GPCR Consortium, CA92078 San Marcos, USA

g, School of Pharmacy, Fudan University, 201203, Shanghai, China

‡: These authors contributed equally to this work

*: Corresponding authors: Prof. Dr. M-W. Wang, E-mail: mwwang@simm.ac.cn
Prof. Dr. W Shui, E-mail: shuiwq@shanghaitech.edu.cn

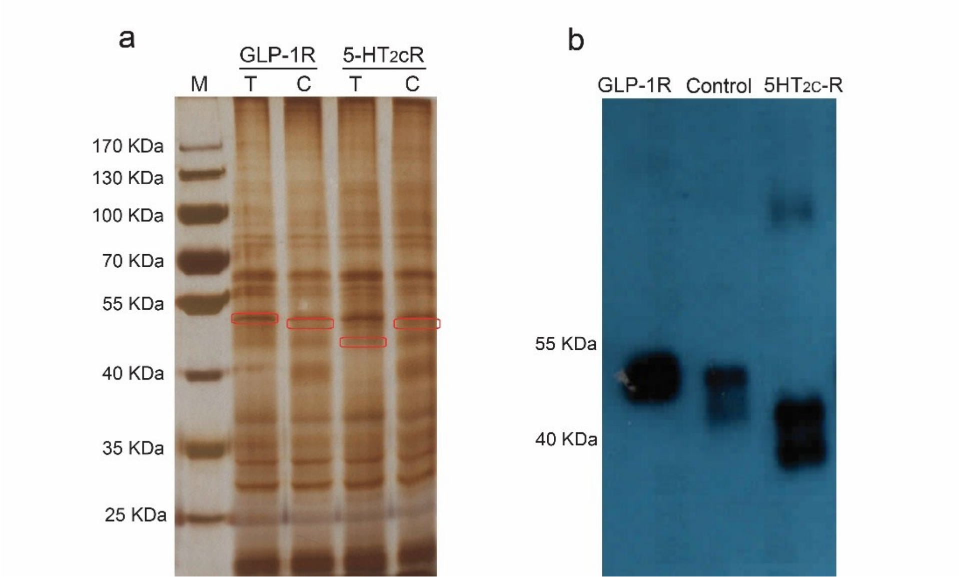

Figure S1. (a) SDS-PAGE image of proteins extracted from membrane fractions used in GLP-1R or 5-HT2CR ligand screening experiments. T and C represent cell membranes expressing the receptor target and the control (rhodopsin), respectively. The positions of target and control proteins are indicated by red squares. (b) Western blot of two protein targets and the control (rhodopsin) expressed in the cell membranes detected by anti-His antibody.

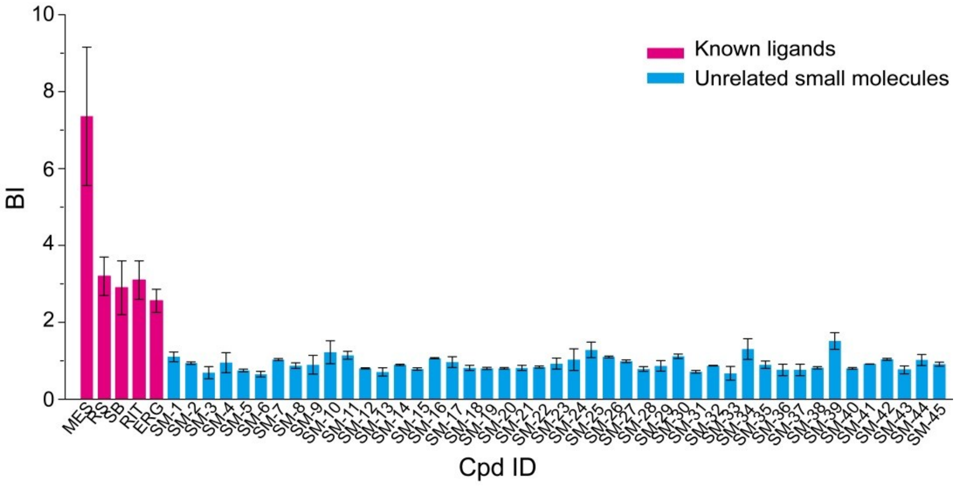

Figure S2. Ligand identification from a 50-compound mixture by affinity MS screening against 5-HT2CR. BI values are shown as mean and s.d. of four individual assays. Five known ligands (MES, mesulergine; RS, RS102221; SB, SB206553; RIT, ritanserin; and ERG, ergotamine) were all identified with BI >2. The remaining small molecules in the mixture were randomly picked from an in-house library and had no documented connection to the receptor.

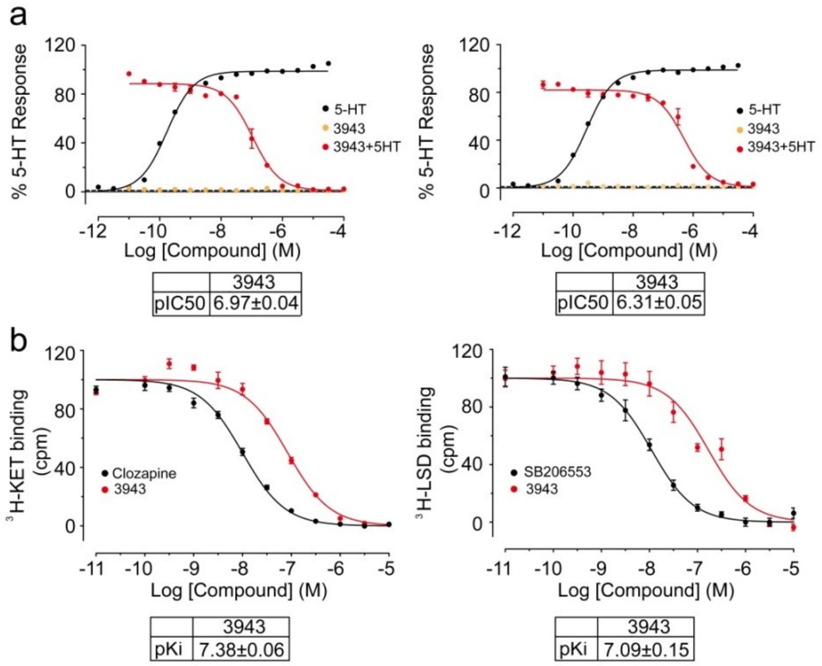

Figure S3. (a) Inhibition of 5HT2AR (left) and 5HT2BR (right) activation by 3943 revealed by the calcium mobilization assay. (b) Affinity of 3943 to 5HT2AR (left) and 5HT2BR (right) determined by the radioligand binding assay. Clozapine and SB 206553 are positive controls. Measurement of pKi and pIC50 is represented by mean and s.d. of triplicate measurements.

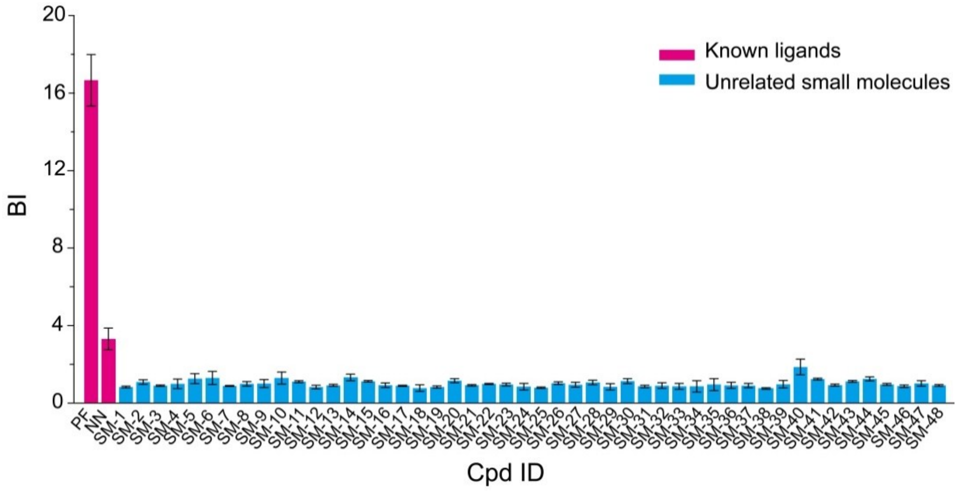

Figure S4. Ligand identification from a 50-compound mixture by affinity MS screening against GLP-1R. BI values are shown as mean and s.d. of four individual assays. Two known ligands (PF, PF06372222; NN, NNC0640) were identified with BI >2. The rest of small molecules in the mixture were randomly picked from an in-house library and had no documented connection to the receptor.

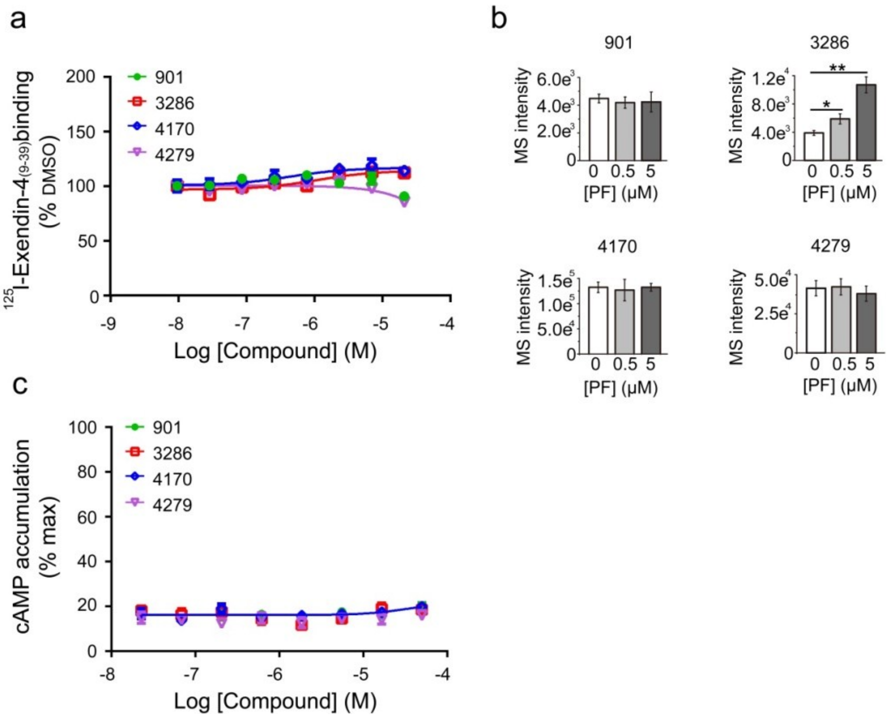

Figure S5. (a) Receptor binding assay showed no effects of any test ligands on modulation of the radiolabeled exendin-4(9-39) binding to GLP-1R. (b) The affinity MS assay showed none of the test ligands could be directly competed off from the receptor by increasing the amount of NAM PF06372222 (PF). In this assay, the GLP-1R TMD-expressing membranes were incubated with the 4-ligand mixture (each at 50 nM) or a mixture of four ligands (each at 50 nM) plus PF06372222 (at 500 nM or 5 μM). The MS intensity of each ligand detected in the target incubation sample was used to compare binding capacity of the ligand under different conditions. 3286 showed enhanced binding to the receptor whereas other ligands did not change their binding properties with increased amounts of PF06372222. MS intensity of each ligand is shown as mean and s.d. of three individual assays. Statistical analysis was performed using two-tailed homoscedastic t-tests (*p <0.05, **p <0.01). (c) No stimulation of cAMP production was observed in GCGR-expressing cells treated with any of the test ligands at varying concentrations in the presence of glucagon (0.02 nM).

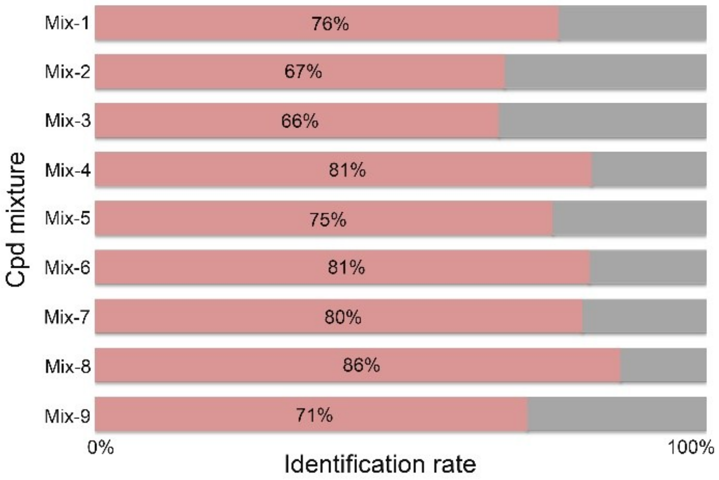

Figure S6. Compound identification rates of 9 cocktails using the LC-MS method. Compounds were identified based on accurate mass measurement (<10 ppm) and isotope envelop matching (<20% deviation).

Note: Table S1 and Table S4 are provided in separate Excel sheets due to their large size.

Table S2. Properties of 12 known ligands to 5-HT2C and 5-HT2A receptors identified in the primary screening.

Compound	Name	BI[a]	ΔT_m (°C)[b]	Bioactivity[c]	Ki (nM)[c]
418	Paliperidone	9.7±3.6	17.6, 19.9	5-HT_{2A} antagonist	0.4-1.2
1216	Mianserin	8.9±1.5	15.1, 16.1	5-HT_{2C} antagonist	0.6 -8.1
446	Pizotyline	8.8±1.4	19.3, 16.6	5-HT_{2C} antagonist	1.4
768	Ziprasidone	6.5±0.8	2.3, 1.9	5-HT_{2C} antagonist	0.6-1.3
265	Clozapine	4.2±1.0	14.9, 17.1	5-HT_{2C} antagonist	2.3-73
2811	Asenapine	4.3±0.5	8.8, 11.5	5-HT_{2C} antagonist	0.9-2.5
1130	Cyproheptadine	3.7±0.3	14.8, 13.9	5-HT_{2C} antagonist	1.3
1248	Ritanserin	3.5±0.5	20.0, 17.1	5-HT_{2A} antagonist	0.6-36
1152	Risperidone	3.3±0.5	16.1, 16.3	5-HT_{2C} antagonist	6.4-63
1449	Metergoline	3.2±0.4	8.6, 11.5	5-HT_{2C} antagonist	0.5
1108	Pirenperone	2.3±0.4	14.9, 16.6	5-HT_{2C} antagonist	60.0
884	Loxapine	2.2±0.3	14.4, 17.1	5-HT_{2A} antagonist	2.4

[a] BI determined in the primary affinity MS screening. [b] T_m shift of each compound compared to the apo receptor in the thermal shift assay (data from two replicates). The concentration of each ligand was 100 μM. ΔT_m >5°C indicates significant increase of the receptor thermostability induced by the compound. [c] Data from ChEMBL and Binding DB databases.

Table S3. Properties of 4 unknown ligands validated in the second-round affinity MS assay.

Compound	Name	BI[a]	ΔT_m (°C) [b]	Bioactivity[c]
3943	AT-56	3.2±0.5	6.2, 6.9	Prostaglandin D_2 synthase Inhibitor
3931	AT 7519	2.7±0.5	-3.0,-0.2	Cell Cycle/checkpoint, CDK inhibitor
462	Ritonavir	2.5±0.3	0.2, 0.2	Antiviral activity, HIV protease inhibitor
1382	Orbifloxacin	2.4±0.5	2.0, 2.0	Antibacterial activity

[a] BI determined in the validation affinity MS assay. [b] T_m shift of each compound compared to the apo receptor in the thermal shift assay (data from two replicates). The concentration of each ligand was 100 μM. ΔT_m >5°C indicates significant increase of the receptor thermostability induced by the compound. [c] Data from ChEMBL database and literature.

Table S5. Enhanced GLP-1 radioligand binding and cAMP production in GLP-1R-expressing cells treated by a specific ligand.

	**901**	**3286**	**4170**	**4279**
^{125}I-GLP-1 binding assay[a]	4.12±0.19	5.2±0.16	5.41±0.29	5.08±0.26
cAMP assay[a]	5.37±0.25	5.19±0.04	4.65±0.43	5.45±0.05

[a] Data shown are mean and s.e.m. of pEC_{50} values determined in experimental triplicates. The corresponding titration curves are shown in Figs. 3c and 3d.
